# Current Status and Future Perspectives of Robotic Surgery for Esophagogastric Cancer in Asia

**DOI:** 10.1002/ags3.70266

**Published:** 2026-08-17

**Authors:** Jia Jun Ang, Jimmy Bok Yan So

**Affiliations:** ^1^ Department of Surgery University Surgical Cluster, National University Health System Singapore Singapore; ^2^ Department of Surgical Oncology National University Cancer Institute National University Health System Singapore Singapore; ^3^ Yong Loo Lin School of Medicine National University of Singapore Singapore Singapore

## Abstract

Robotic‐assisted surgery (RAS) has moved from an experimental adjunct to an increasingly established component of gastrointestinal (GI) oncological practice in parts of Asia, a region that carries a disproportionately high burden of gastric and esophageal cancer. Asian surgeons have been among the earliest and most prolific contributors to the clinical evidence base for robotic gastrectomy and robot‐assisted minimally invasive esophagectomy. This narrative review synthesizes evidence from randomized trials (including the ROBOT, RAMIE, and REVATE trials, and the ongoing MONA LISA [JCOG1907] study), multicenter prospective and propensity‐matched cohort studies, and recent meta‐analyses to critically appraise the efficacy, safety, cost, and future trajectory of RAS in Asia. Across multiple Korean, Japanese, and Chinese series, RG and RAMIE are associated with reduced short‐term morbidity, improved lymph node retrieval in technically demanding anatomic regions, and lower rates of recurrent laryngeal nerve injury compared with conventional laparoscopic or thoracoscopic approaches; however, much of the evidence on long‐term oncological outcomes remains retrospective or derived from propensity‐score analyses rather than mature randomized data, and results across studies are not always concordant. We argue that robotic surgery in Asia is best understood as passing through three overlapping transitions: technical and oncological validation, economic and educational democratization, and digital and telesurgical integration, each of which carries distinct and unresolved questions. We conclude that while RAS has demonstrably reshaped upper GI surgical practice in high‐volume Asian centers, its designation as a definitive regional standard of care should await longer‐term randomized outcomes, standardized credentialing, and more equitable deployment across the region.

## Abstract 2

This article is a narrative review of current evidence of robotic surgery for esophagogastric surgery in Asia. We have evaluated robotic surgery on the basis of technical and oncological validation, economic and educational democratization, and digital and telesurgical integration. We hope to contribute to understanding of robotic surgery in this context by summarizing current evidence and aid with future directions of research.

## 1 Introduction

The surgical management of gastrointestinal (GI) malignancies has undergone a substantial transformation over the last three decades. The shift from open surgery to minimally invasive surgery (MIS) was driven by efforts to reduce surgical trauma and accelerate recovery. Conventional laparoscopy, however, is associated with ergonomic and technical constraints, including two‐dimensional visualization and instruments with restricted degrees of freedom.

The introduction of the Da Vinci Surgical System (DVSS) in 1995 (FDA‐approved in 2000) addressed many of these limitations by providing three‐dimensional high‐definition visualization, wristed instruments with seven degrees of freedom, and tremor filtration [1]. In Asia, where the incidence of gastric and esophageal cancer is among the highest in the world and complex oncological resections are correspondingly frequent, has been a major contributor to the clinical evidence base for robotic surgery over the past decade [2]. This has been accompanied by the development of indigenous robotic platforms, the establishment of structured training paradigms, and a substantial body of clinical evidence, much of it generated within Asian centers [3, 4].

For this narrative review, relevant literature was identified through searches of PubMed/MEDLINE for articles published mainly between 2015 and 2026, using combinations of the terms ‘robotic surgery’, ‘robotic gastrectomy’, ‘robot‐assisted minimally invasive esophagectomy’, ‘gastric cancer’, ‘esophageal cancer’, ‘learning curve’, ‘cost‐effectiveness’, and ‘Asia’. Priority was given to randomized controlled trials, multicenter prospective studies, and large propensity‐score‐matched cohorts; the reference lists of retrieved articles and recent systematic reviews were hand‐searched to identify additional pertinent studies. The selection and interpretation of studies reflect the authors' judgment of clinical relevance to contemporary Asian practice, and this inherent selectivity is acknowledged as a limitation of the present review.

Beyond cataloging individual trials, this review is organized around an explicit argument: that the trajectory of robotic surgery for esophagogastric cancer in Asia can be understood as several overlapping and still incompletely resolved, transitions. Firstly, technical and oncological validation against laparoscopic and open benchmarks. Secondly, economic and educational democratization of access across markedly heterogeneous health systems, and lastly digital integration through artificial intelligence and telesurgery. By framing evidence in this manner, we hope to identify the specific gaps in evidence and practice that will determine whether robotic surgery becomes a genuinely regional standard of care or remains concentrated in a small number of high‐volume centers.

## 2 Gastric Cancer

The clinical validation of robotic gastrectomy (RG) in Asia has advanced considerably, particularly in Korea, Japan, and China, although much of the long‐term evidence supporting its adoption is derived from single‐ or multi‐institutional cohort studies rather than randomized trials. South Korea has been at the forefront of robotic gastrectomy for gastric cancer since its earliest adoption. An early case series reporting the initial experience with robotic distal subtotal gastrectomy with D2 dissection from 2005 to 2009 demonstrated a mean lymph node retrieval of 41.8 nodes, a complication rate of 11.6%, and 5‐year overall and relapse‐free survival of 92.8% and 91.8%, respectively [5]. A multicenter prospective study by Kim et al. comparing 223 robotic and 211 laparoscopic gastrectomies (LG) showed similar overall complication rates (RG 11.9% vs. LG 10.3%, *p* = 0.619) and identical major complication rates (RG 1.1% vs. LG 1.1%, *p* = 0.999), with no operative mortality [6]. This was associated; however, with significantly longer operative times (RG 221 vs. LG 178 min, *p* = 0.001) and higher costs (RG $13 432 vs. LG $8090, *p* = 0.001) [6]. The maturation of robotic gastrectomy was subsequently illustrated in a landmark series of 2000 consecutive cases performed over 14 years (2005–2019) from Severance Hospital, Korea. Major complication and mortality rates were 3.1% and 0.3%, respectively, with 5‐year overall survival rates of 97.6%, 91.9%, and 69.2% for stages I, II, and III. Notably, the proportion of technically demanding procedures, such as total and proximal gastrectomies, increased over time, reflecting growing confidence in the robotic platform for complex resections [7].

In Japan, a multi‐institutional prospective single‐arm study led by Uyama et al. (*n* = 330) further demonstrated the safety of RG, with a morbidity rate (Clavien‐Dindo grade ≥ 3a) of only 2.5%, compared with 6.4% in a historical laparoscopic cohort (*p* = 0.0018) [8]. This study contributed to the subsequent inclusion of robotic gastrectomy within Japan's national medical insurance coverage. A randomized clinical trial conducted by Ojima et al. comparing the short‐term outcomes of RG versus LG found that overall complications of Grade II or higher were significantly lower in the robotic group (8.5% vs. 19.3%, *p* = 0.019), with no postoperative pancreatic fistula observed in the robotic cohort, supporting the feasibility and safety of the robotic approach for gastrectomy [9]. This finding was complemented by a landmark randomized controlled trial from China by Lu et al., enrolling 300 patients randomized to robotic versus laparoscopic distal gastrectomy [10]. The modified intention‐to‐treat analysis of 283 patients demonstrated that robotic distal gastrectomy was associated with reduced postoperative morbidity (9.2% vs. 17.6%, *p* = 0.039), faster postoperative recovery, and a milder inflammatory response. Patients in the robotic group were also more likely to initiate adjuvant chemotherapy earlier (median 28 vs. 32 days, *p* = 0.003), a finding of potential, though as yet unconfirmed, oncologic significance [10].

One advantage demonstrated for robotic gastrectomy is improved lymph node retrieval, particularly in technically challenging anatomic regions. A Chinese multicenter study of 5402 patients found that robotic gastrectomy retrieved significantly more lymph nodes, both in total (32.5 vs. 30.7, *p* = 0.001) and specifically in suprapancreatic areas (13.3 vs. 11.6, *p* = 0.001) [11]. In a prospective randomized controlled study from China using objective clinical human reliability analysis (OCHRA) to analyse unedited surgical videos for technical performance, the highest number of technical errors occurred during suprapancreatic lymph node dissection in both robotic and laparoscopic approaches (7.29 ± 1.88 vs. 9.43 ± 2.24, *p* = 0.001). Robotic total gastrectomy was associated with significantly fewer total technical errors (46.11 ± 5.63 vs. 58.79 ± 8.45, *p* = 0.001) and a higher lymph node yield at the upper margin of the pancreas (13.32 ± 4.17 vs. 9.36 ± 3.81, *p* = 0.001) [12].

Whether these technical advantages translate into superior oncological outcomes remains less certain. A multi‐institutional retrospective comparative study using propensity score matching (PSM), reported by Suda et al., found that RG for clinical Stage I/II gastric cancer was associated with significantly higher 3‐year overall survival (OS) and recurrence‐free survival (RFS) than LG (*p* < 0.001) [13]. By contrast, a propensity score‐weighted analysis of 2084 patients comparing robotic and laparoscopic gastrectomy from Korea demonstrated comparable 5‐year overall survival (93.2% vs. 94.2%, HR 0.88, *p* = 0.636) and recurrence‐free survival (95.3% vs. 96.3%, HR 1.24, *p* = 0.498), with reduced intraoperative blood loss in the robotic group [14].

An extended analysis of the randomized Phase 2 trial from China published by Lu et al., comparing robotic distal gastrectomy (RDG) with laparoscopic distal gastrectomy (LDG), found a higher 3‐year disease‐free survival (DFS) rate in the robotic group (85.8% vs. 73.2%, *p* = 0.011), an effect the authors attributed largely to more precise D2 lymph node dissection in the suprapancreatic area [15]. As this remains the only adequately powered randomized trial to report a survival endpoint favoring RG, corroboration from other randomized studies is required before this finding can be considered generalizable.

The MONA LISA study (JCOG1907) is a multicenter, randomized controlled Phase III trial conducted by the Japan Clinical Oncology Group (JCOG) across 46 Japanese institutions, designed to investigate the superiority of robot‐assisted gastrectomy (RG) over laparoscopic gastrectomy (LG) for gastric cancer. The trial, which commenced enrolment in March 2020, is the first large‐scale multicenter randomized controlled trial to directly compare these two minimally invasive approaches with a superiority design focused on safety; its primary endpoint is the incidence of postoperative intra‐abdominal infectious complications of Clavien‐Dindo Grade II or higher [16]. Together with mature follow‐up from the Chinese RCT by Lu et al., MONA LISA is expected to provide the level of evidence needed to determine whether the technical advantages of robotic gastrectomy translate into a genuine outcome benefit, or whether the more favorable signals reported to date reflect selection and confounding inherent to non‐randomized comparisons [15, 16].

## 3 Esophageal Cancer

Esophagectomy remains one of the most technically demanding procedures in gastrointestinal surgery. When evaluating the comparative evidence for robot‐assisted minimally invasive esophagectomy (RAMIE), it is critical to distinguish between two distinct clinical questions that are frequently conflated: its performance relative to open esophagectomy, and its performance relative to conventional, non‐robotic minimally invasive esophagectomy (MIE).

### 3.1 RAMIE Versus Open Esophagectomy

The ROBOT trial (*n* = 112), a Dutch randomized controlled trial comparing RAMIE with open surgery, found that overall surgery‐related postoperative complications were significantly less frequent after RAMIE (59% vs. 80%; RR 0.74; 95% CI, 0.57–0.96; *p* = 0.02). The robotic cohort also demonstrated fewer cardiopulmonary complications, reduced postoperative discomfort, and improved short‐term functional recovery and health‐related quality of life [17]. While this trial established the feasibility and short‐term superiority of RAMIE over open transthoracic esophagectomy, it did not address whether the robotic platform offers an incremental benefit over conventional thoracoscopic or laparoscopic MIE. This latter comparison perhaps holds a greater relevance for contemporary practice, particularly in Asian centers where conventional MIE is already the established standard of care.

### 3.2 RAMIE Versus Conventional MIE

The RAMIE trial (*n* = 362) by Yang et al. from China demonstrated that, compared with conventional MIE, RAMIE improved the thoracic lymph node yield in patients who received neoadjuvant treatment (median 15 vs. 12 nodes, *p* = 0.016) and the rate of left recurrent laryngeal nerve (RLN) lymph node dissection (79.5% vs. 67.6%, *p* = 0.001), and was associated with a shorter operative time (203.8 vs. 244.9 min, *p* < 0.001). Short‐term outcomes including blood loss, conversion rate, R0 resection, 90‐day mortality, and the rate of major complications were comparable between groups [18].

The REVATE trial provided further evidence for nerve preservation specifically in the comparison with conventional MIE. In this multicenter randomized controlled trial from Taiwan, robot‐assisted thoracoscopic esophagectomy was compared with video‐assisted thoracoscopic esophagectomy. RAMIE was associated with a significantly lower rate of permanent RLN palsy at 6 months (5.8% for RAMIE vs. 20% for MIE, *p* = 0.003), consistent with a role for three‐dimensional visualization and precise instrumentation in protecting vulnerable structures during mediastinal dissection [19].

With respect to long‐term oncological outcomes for RAMIE versus conventional MIE, a multicenter propensity score‐matched cohort study from Japan, including 268 of 396 patients (134 matched pairs) across six hospitals, compared short‐ and long‐term outcomes of RAMIE versus MIE for esophageal cancer. The 3‐ and 5‐year overall survival rates were 77% and 66% for RAMIE versus 74% and 66% for MIE (HR 0.89; 95% CI 0.57–1.37; *p* = 0.59), with similar relapse‐free survival between groups [20]. The primary survival endpoint of the Chinese RAMIE trial remains immature and is awaited with interest [18].

Interpreted together, the available randomized and propensity‐matched evidence indicates that, relative to conventional MIE, RAMIE reduces recurrent laryngeal nerve injury and, in some series, improves lymph node yield, without a demonstrable difference in short‐term mortality, morbidity, or long‐term survival.

## 4 Current Trends and Market Competition in Asia

Japan has seen a marked increase in robotic procedures following National Health Insurance reimbursement in April 2018. A nationwide study by Nishigori et al. reported that the monthly number of robotic surgeries nearly doubled immediately following reimbursement [2]. By 2022, the Japanese government further increased remuneration for robotic gastrectomy, encouraging wider adoption.

While the Da Vinci system remains the market leader, new platforms are emerging to challenge its dominance, including the Hinotori surgical robot system (Medicaroid, Japan) [21, 22, 23], REVO‐I (Meere Company, South Korea) [24], the Sentire Surgical System (Cornerstone Robotics, Hong Kong, China) [25, 26], Hugo RAS (Medtronic, USA) [27], and Versius (CMR Surgical, UK) [28]. The emergence of these competing platforms is expected to exert downward pressure on costs over time, although direct comparative data on their clinical performance relative to the Da Vinci system remain limited for upper GI oncological indications specifically, and their long‐term role in this setting has yet to be established.

## 5 Learning Curve and Educational Standardization

The learning curve for robotic gastrectomy is generally reported to be shorter than for laparoscopic gastrectomy. A multicenter trial from South Korea found that 25 cases were needed to reach proficiency, with an additional 23 cases needed for mastery [29]. For RAMIE, reported learning curves plateau at 20 to 85 cases, depending on the outcome measure and surgical approach used [30, 31]. Mastery of the mediastinal phase of RAMIE has been shown to improve lymph node yield and reduce pneumonia rates [32].

These learning‐curve estimates; however, are drawn predominantly from high‐volume academic centers in Korea and Japan with structured proctorship and defined case‐volume thresholds, their generalizability to lower‐volume or less‐resourced institutions elsewhere in Asia is uncertain. Formal credentialing pathways for robotic upper GI surgery remain inconsistent across the region and in the absence of standardized case‐volume requirements or mandatory proctorship, outcomes achieved during the early adoption phase at other institutions may not reliably mirror those reported from the centers that generated the learning‐curve data summarized above [29, 30, 31, 32].

## 6 Limitations and Barriers to Equitable Adoption

Several limitations temper enthusiasm for robotic surgery as the standard of care for esophagogastric cancer in Asia. First, the Level I evidence base remains incomplete. Outside of the ROBOT, RAMIE, and REVATE trials for esophageal cancer and the trials by Ojima et al. and Lu et al. for gastric cancer, much of the data on long‐term oncological outcomes, particularly overall and disease‐specific survival, are derived from retrospective or propensity‐matched cohorts rather than adequately powered randomized trials. Furthermore, as noted above, results across these cohorts are not always concordant. The MONA LISA trial and mature follow‐up of the Chinese RAMIE trial are expected to substantially narrow this gap, but their results are not yet available [16, 18].

Second, access to robotic platforms remains highly uneven across Asia. National reimbursement policies, such as Japan's 2018 insurance coverage for robotic gastrectomy, have been transformative in high‐income health systems, but comparable coverage is largely absent in lower‐ and middle‐income countries in South and Southeast Asia, where high capital and disposable costs continue to restrict robotic surgery to a limited number of tertiary referral centers [2, 33]. The survival and quality‐of‐life benefits documented in Korean, Japanese, and Chinese tertiary centers may not, therefore, be representative of outcomes achievable more broadly across the region.

Third, as outlined above, credentialing and case‐volume requirements for robotic upper GI surgery are not standardized across Asian jurisdictions, and outcome auditing during early adoption is inconsistently mandated. Finally, most of the evidence reviewed here originates from a comparatively small number of high‐volume academic institutions in East Asia; data from South Asia, Southeast Asia, and the Middle East remain sparse by comparison, which limits the generalizability of the conclusions drawn in this review to the whole of the Asian region.

## 7 Overcoming the Economic Barrier

Cost‐effectiveness remains a primary hurdle for RAS. Direct costs include capital acquisition of the robotic system and disposable instruments. In South Korea, robotic gastrectomy was significantly more expensive than laparoscopic gastrectomy ($13 432 vs. $8090) [33]. A trial‐based economic evaluation from China reported an incremental cost‐effectiveness ratio (ICER) of $85739.73 per quality‐adjusted life‐year (QALY), which exceeded three times the per capita GDP in China, suggesting that cost‐effectiveness by conventional willingness‐to‐pay thresholds is currently achieved primarily in higher‐income healthcare systems [34].

Further analyses suggest that, while intraoperative costs for RG are significantly higher than for open or laparoscopic surgery, reduced postoperative hospitalization costs may help narrow this gap [35]. Market competition from emerging Asian robotic companies may, over time, help reduce the cost of consumables and hardware. However, whether this will be sufficient to make RAS a genuinely universal standard of care, rather than one concentrated in better‐resourced centers or health systems, remains to be seen.

## 8 Future Perspectives: AI And Telesurgery

Digital integration represents a further but still largely investigational frontier for RAS in Asia. AI‐driven platforms may, in time, allow for the collection of granular surgical data, facilitating objective performance metrics and automated training. At present; however, such applications remain predominantly at the research and skill‐assessment stage rather than in routine clinical deployment.

A notable recent advance in this domain is the demonstration of long‐range telesurgery. In a preclinical study, surgeons from the National University Hospital (Singapore) and Fujita Health University (Japan) performed three robotic radical D2 gastrectomies on live porcine models in Japan while located approximately 5000 km away in Singapore. Using the Hinotori system and high‐speed fiber‐optic connections, the procedures were completed in under 205 min without intraoperative complications, suggesting that at least in a controlled preclinical setting, geographic distance need not preclude the delivery of specialized surgical expertise [36]. Translating this feasibility work into routine human clinical practice will require solutions to substantial regulatory, medicolegal, and connectivity‐redundancy challenges that will require addressing.

## 9 Conclusion

Robotic surgery for esophagogastric cancer in Asia is best characterized not as a completed transition but as an evolving and still incompletely validated shift in surgical practice. The evidence reviewed here supports RG and RAMIE as safe and technically advantageous alternatives to conventional laparoscopic and thoracoscopic surgery in high‐volume centers, with reproducible benefits in lymph node retrieval and nerve preservation. Whether these technical advantages translate into superior long‐term oncological outcomes remains an open question that hopefully the maturation of the MONA LISA trial, the Chinese RAMIE trial, and comparable regional randomized studies can resolve. For surgeons and health systems across Asia, the more immediate priorities may be structural rather than purely technological. Standardizing credentialing and case‐volume requirements, extending reimbursement and infrastructure beyond a small number of tertiary centers, and building regional registries capable of capturing outcomes across the full spectrum of institutions rather than only early‐adopting, high‐volume centers. Advances in AI‐assisted training and telesurgery may eventually help address some of these disparities, but their translation from preclinical feasibility to routine clinical use remains at an early stage. The task ahead for the Asian upper GI surgical community is therefore twofold: to complete the randomized evidence base begun by trials such as MONA LISA and the Chinese RAMIE trial, and to ensure that the benefits of robotic surgery are deployed equitably.

## Author Contributions

**Jia Jun Ang:** writing – original draft. **Jimmy Bok Yan So:** writing – review and editing, supervision.

## Funding

The authors have nothing to report.

## Disclosure

The authors have nothing to report.

## Conflicts of Interest

The authors declare no conflicts of interest.

## Data Availability

Data sharing not applicable to this article as no datasets were generated or analysed during the current study.
